# Supplementary material for: Electron–electron interactions and the paired-to-nematic quantum phase transition in the second Landau level
Source: Nat Commun. 2018 Jun 19;9:2400. doi: 10.1038/s41467-018-04879-1 (PMC6008478; doi:10.1038/s41467-018-04879-1)
Supplement: Supplementary file 1 — Supplementary Information [file 41467_2018_4879_MOESM1_ESM.pdf]

Electron-Electron Interactions and the Paired-to-Nematic Quantum Phase Transition in the Second Landau Level

K.A. Schreiber, N. Samkharadze, G.C. Gardner, Y. Lyanda-Geller,

M.J. Manfra, L.N. Pfeiffer, K.W. West, G.A. Csáthy

Supplementary Note 1: Evolution of sample parameters with pressure.
Hydrostatic pressure has the effect of changing many sample parameters, most significantly electron effective mass, dielectric constant, g-factor, and carrier density [3-7]. In order to calculate Landau level mixing, we needed an estimate of effective mass and dielectric constant. We used equations taken from Ref. [4]: $m(P)/m(0) = 1 + 0.007P$ for effective mass, and $\varepsilon(P)/\varepsilon(0) = 1 - 0.0017P$ for dielectric constant, where P is measured in kbar. We use $\varepsilon(P = 0) = 12.9$ and $m(P = 0) = 0.067m_e$, the values in GaAs.

We clearly observed the decrease of electron density in sample A, which changes linearly [3, 7]. We obtain the experimental fit $n(P) = 29.24 – 2.17P$, where P is measured in kbar and n is measured in units of 10^{10} cm^{-2}. In order to measure the electron density at each pressure step, we took the magnetic field at the FQHS minima in longitudinal resistance R_{xx}, for several fractions that display very narrow minima. These include the narrow minima at $\nu = 11/5$, $14/5$, and $11/7$. These magnetic fields then yield the density through the definition of the filling factor, $\nu = hn/eB$.

Supplementary Note 2: Destruction of the anisotropic phase at the highest pressures.
In Supplementary Figure 4 we show the disappearance of the nematic behavior at high values of the pressure. Based on data in the literature, in a sample of a given density, the nematic is less prominent at lower magnetic fields, i.e. at higher filling factors. Data shown is consistent with this observation. At P=10.15 kbar we observe clearly noticeable nematic phases at ν=5/2 and 7/2, but only a remnant of a nematic at ν=9/2. At an even higher pressure of P=10.54 kbar, all nematic phases are significantly weakened. We think that this trend of the data, when considered together with the very low magnetic fields near 1 T, is consistent with a disorder driven destruction of the nematic at such large pressures.

Supplementary Note 3: Details pertaining to the measurement environment of sample B. This sample was measured in a ^{3}He immersion cell. Therefore the actual pressure at which the measurements were taken was $P \sim 0.1$ bar, the vapor pressure of the liquid-gas interface of the ^{3}He in the capillary tube used for filling the immersion cell, located just above the 1 K pot level. This value is different from that of setups in which the sample is in vacuum. However, this pressure is negligible as compared to the kbar level pressures in our pressure cell. Furthermore, we note that the resistance anisotropy of sample B at ν=7/2, shown in Fig.6 of the main text, greatly exceeds that in sample A shown in Fig.2, because of different electronic temperatures. Indeed, in the immersion cell we reached $T \sim 4.5$ mK electronic temperature, which is significantly lower than the estimated $T \sim 12$ mK achieved in the pressure cell.

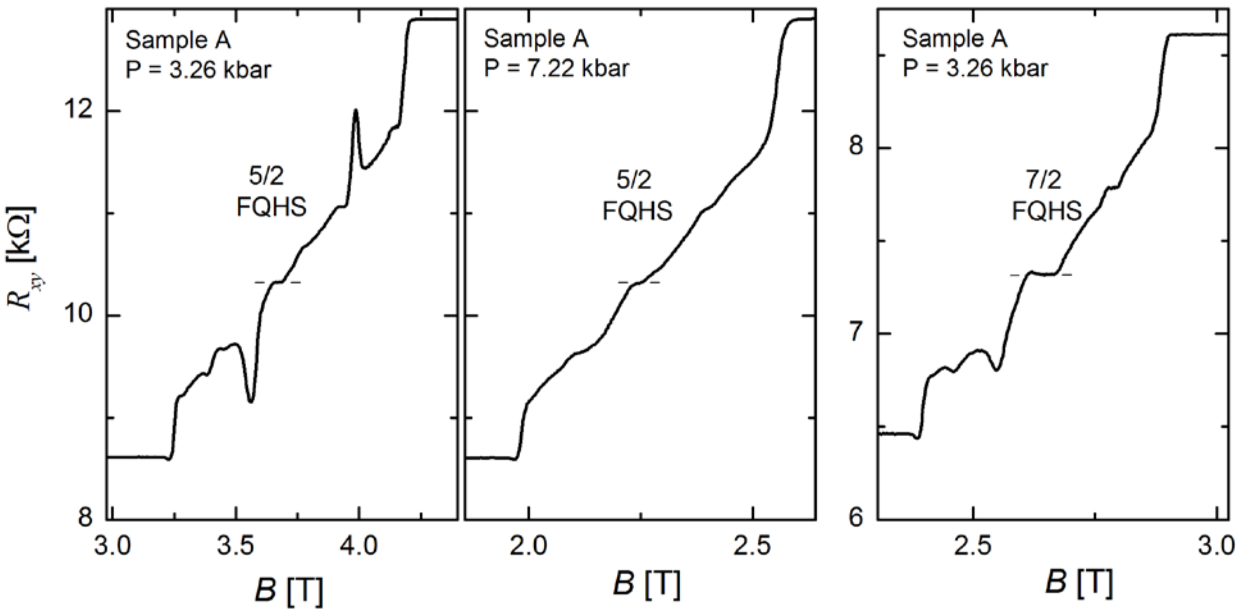

Supplementary Figure 1: The Hall resistance measured at T ~ 12 mK at two pressures in the pressurized sample A, showing the quantized resistance of the FQHSs at ν = 5/2 and ν = 7/2. The first two panels (left and center) show the region of filling factors around ν = 5/2 at P = 3.26 and P = 7.22 kbar, corresponding to Fig.2a and Fig.2b from the main text. The third panel (right) shows the region of filling factors around ν = 7/2, corresponding to Fig.2a from the main text.

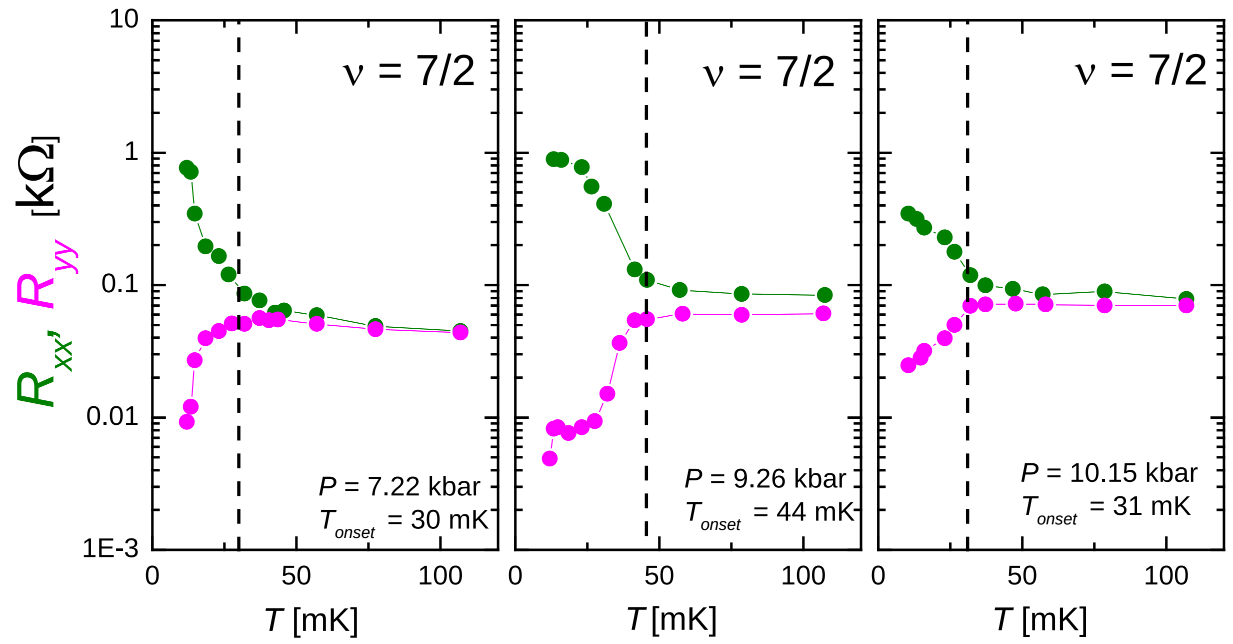

Supplementary Figure 2: Temperature dependence of the magnetoresistance measured at ν = 7/2 along two mutually perpendicular crystallographic directions of GaAs. Data is for Sample A. The vertical dashed lines indicate the onset temperature T_{onset}, defined as the temperature at which R_{xx} = $2R_{yy}$.

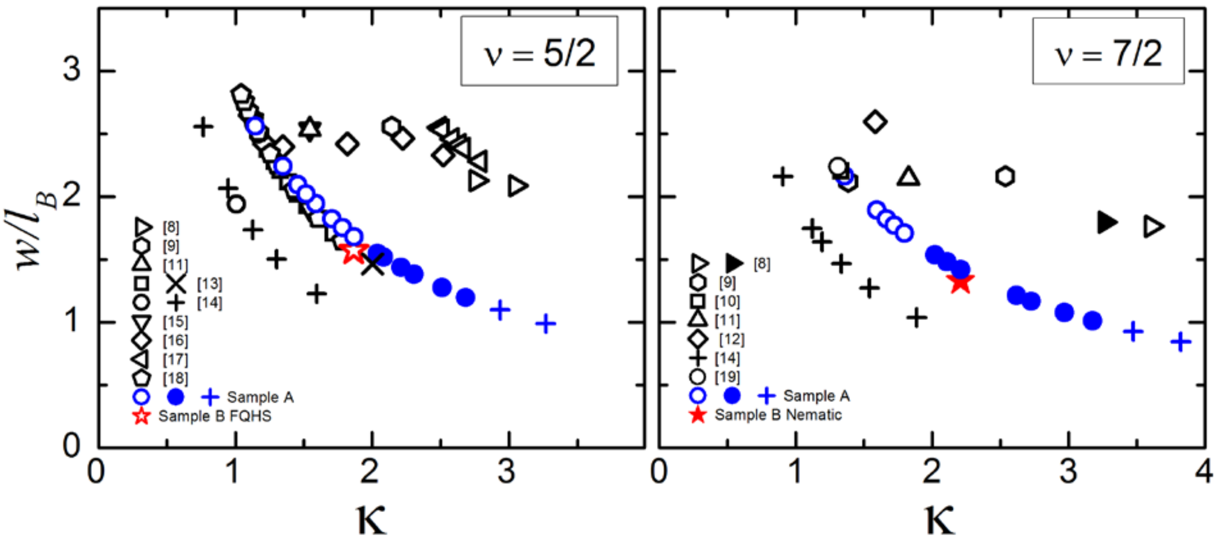

Supplementary Figure 3: The κ-w/l_B space sampled in the second orbital Landau level. Blue symbols are for our pressurized sample A, red ones for our sample B measured in the ambient, and black symbols are for samples from the literature, measured also in the ambient. Open symbols represent fractional quantum Hall states, closed ones nematics. The **+** and x symbols are states where neither a FQHS nor a nematic phase are demonstrated.

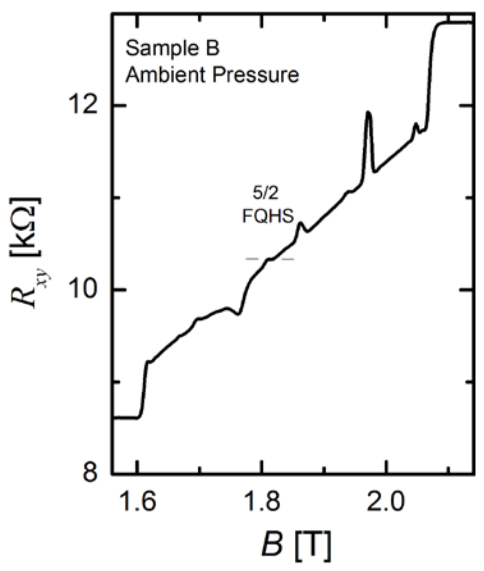

Supplementary Figure 4: Hall resistance in the unpressurized sample B around ν = 5/2, showing the quantized resistance of a FQHS. The temperature is about T ~ 12 mK.

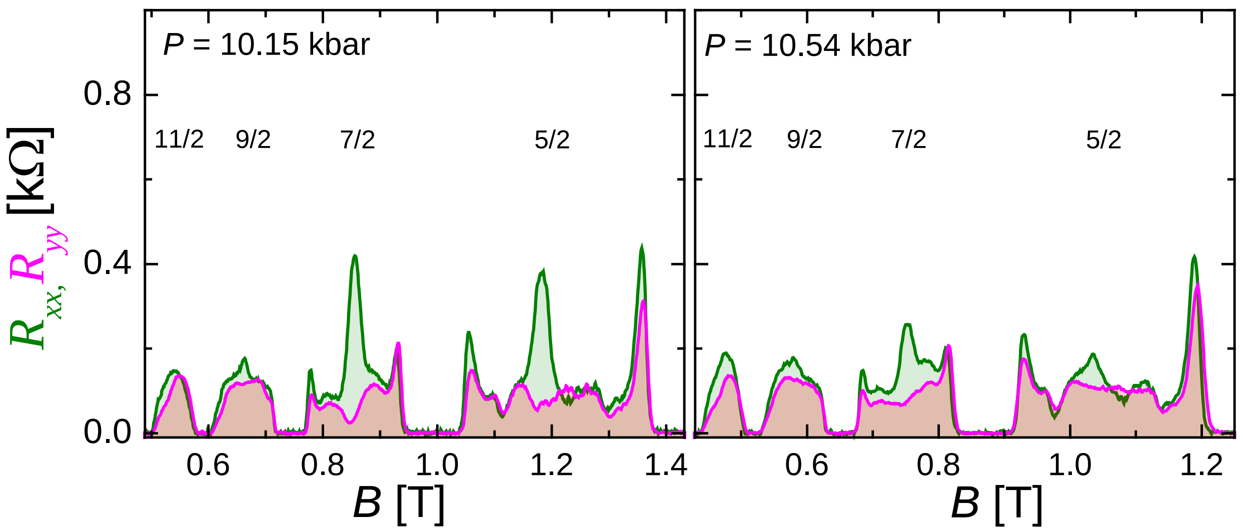

Supplementary Figure 5: Details of the nematic at ν = 5/2, 7/2, 9/11, and 11/2 at two different pressures close to the nematic-to Fermi liquid critical pressure. Data is for Sample A.

Supplementary Table 1. Sample A T_{onset} and Δ for ν = 7/2

P [kbar]	3.26	3.99	4.54	5.16	5.78	6.47	6.72	7.22	7.7	9.26	9.57	10.15	10.54	11.0	11.41
n [10^{10} cm^{-2}]	22.2	20.6	19.4	18.1	16.7	15.2	14.7	13.6	12.	9.12	8.44	7.18	6.33	5.33	4.43
$T_{onset;7/2}$ [mK]	-	-	-	-	-	-	17	30	39	46	42	31	21	-	-
$\Delta_{7/2}$ [mK]	20	8	11	9	-	-	-	-	-	-	-	-	-	-	-

Supplementary Table 2. Sample A T_{onset} and Δ for ν = 5/2

P [kbar]	3.2	4.54	5.16	5.78	6.72	7.22	7.72	8.58	8.77	9.26	9.57	10.15	10.54	11.0	11.41
n [10^{10} cm^{-2}]	22.	19.4	18.1	16.7	14.7	13.6	12.5	10.6	10.2	9.12	8.44	7.18	6.33	5.33	4.43
$T_{onset;5/2}$ [mK]	-	-	-	-	-	-	-	24	30	28	29	21	10 (est.)	-	-
$\Delta_{5/2}$ [mK]	13	164	142	92	40	48	39	-	-	-	-	-	-	-	-

Supplementary References

[1] Samkharadze, N., Schreiber, K.A., Gardner, G.C., Manfra, M.J., Fradkin, E., & Csáthy, G.A. Observation of a transition from a topologically ordered to a spontaneously broken symmetry phase. *Nature Phys*. **12**, 191-1195 (2016).

[2] Schreiber, K.A., Samkharadze, N., Gardner, G.C., Biswas, R.R., Manfra, M.J., & Csáthy, G.A. Onset of quantum criticality in the topological-to-nematic transition in a two-dimensional electron gas at filling factor ν =5/2. *Phys. Rev. B*, **96,** 041107 (2017).

[3] Maude, D.K. & Portal, J.C. Parallel transport in low-dimensional semiconductor structures. *Semiconductors and Semimetals,* Vol. 55 (1998).

[4] Wasilewski, Z., & Stradling, R.A. Magneto-optical studies of n-GaAs under high hydrostatic pressure. *Semicond. Sci. Technol*. **1**, 264-274 (1986).

[5] Dmowski, L., & Portal, J.C. Magnetotransport in 2D semiconductor systems under pressure. *Semicond. Sci. Technol.* **4**, 211-217 (1989).

[6] Holmes, S., Maude, D.K., Williams, M.L., Harris, J.J., Portal, J.C., Barnham, K.W.J., & Foxon, C.T. Experimental determination of the transport properties of composite fermions with reduced Lande g-factor. *Semicond. Sci. Technol*. **9**, 1549 (1994).

[7] Gregoris, G., Lavielle, D., Beerens, J., Ben Amor, S., Portal, J.C., & Alexandre, F. Two-dimensional electrons at a GaAs-AlAs heterojunction under hydrostatic pressure. *Semicond. Sci. Technol.* **4**, 317-321. (1989).

[8] Pan, W, Serafin, A., Xia, J.S., Yin, L, Sullivan, N.S., Baldwin, K. W., West, K.W., Pfeiffer, L.N., & Tsui., D.C. Competing quantum Hall phases in the second Landau level in the low-density limit. *Phys. Rev B* **89**, 241302 (2014).

[9] Samkharadze, N., Watson, J.D., Gardner, G.C., Manfra, M.J., Pfeiffer, L.N., West, K.W., & Csáthy, G.A. Quantitative analysis of the disorder broadening and the intrinsic gap for the ν=52 fractional quantum Hall state. *Phys. Rev. B* **84**, 121305 (2011).

[10] Eisenstein, J.P., Cooper, K.B., Pfeiffer, L.N., & West, K.W. Insulating and Fractional Quantum Hall States in the First Excited Landau Level. *Phys. Rev. Lett.* **88**, 076801 (2002).

[11] Dean, C.R., Piot, B.A., Hayden, P., Das Sarma, S., Gervais, G., Pfeiffer, L.N., & West, K.W. Contrasting Behavior of the 5/2 and 7/3 Fractional Quantum Hall Effect in a Tilted Field. *Phys. Rev. Lett.* **101,** 186806, (2008).

[12] Liu, Y., Shabani, J., Kamburov, D., Shayegan, M., Pfeiffer, L.N., West, K. W., & Baldwin, K.W. Evolution of the 7/2 Fractional Quantum Hall State in Two-Subband Systems. *Phys. Rev. Lett.*, **107**, 266802 (2011).

[13] Nuebler, J., Umansky, V., Morf, R., Heiblum, M., von Klitzing, K. & Smet, J. Density dependence of the ν = 5/2 energy gap: Experiment and theory. *Phys. Rev. B* **81**, 035316 (2010).

[14] Shi, X., Pan, W., Baldwin, K. W., West, K. W., Pfeiffer, L.N., & Tsui, D.C. Impact of the modulation doping layer on the ν=5/2 anisotropy. *Phys. Rev. B* **91**, 125308 (2015).

[15] Xia, J., Cvicek, V., Eisenstein, J.P., Pfeiffer, L.N., & West, K.W. Tilt-Induced Anisotropic to Isotropic Phase Transition at ν=5/2. *Phys. Rev. Lett.* **105**, 176807 (2010).

[16] Pan, W., Baldwin, K.W., West, K. W., Pfeiffer, L.N., & Tsui, D.C. Spin Transition in the ν=8/3 Fractional Quantum Hall Effect. *Phys. Rev. Lett.* **108**, 216804 (2012).

[17] Samkharadze, N., Ro, D., Pfeiffer, L.N., West, K.W., & Csáthy, G.A. Observation of an anomalous density-dependent energy gap of the ν = 5/2 fractional quantum Hall state in the low-density regime. *Phys. Rev. B* **96,** 085105 (2017).

[18] Watson, J.D., Csáthy, G.A., & Manfra, M.J. Impact of Heterostructure Design on Transport Properties in the Second Landau Level of In Situ Back-Gated Two-Dimensional Electron Gases. *Phys. Rev. Appl.* **3**, 064004 (2015).
[19] Schmidt, B.A., Bennaceur, K., Bilodeau S., Gervais, G., Pfeiffer, L.N. & West, K.W. Second Landau level fractional quantum Hall effects in the Corbino geometry. *Solid State Commun*. **217**, 1 (2015).
[20] Lilly, M.P., Cooper, K.B., Eisenstein, J.P., Pfeiffer, L.N.& West, K.W. Evidence for an anisotropic state of two-dimensional electrons in high Landau levels. Phys. Rev. Lett. 82, 394-397 (1999).
[21] Du, R.R., Tsui, D.C., Stormer, H.L., Pfeiffer, L.N., Baldwin, K.W. & West, K.W. Strongly anisotropic transport in higher two-dimensional Landau levels. Solid State Commun. 109, 389-394 (1999).
